# Maternal Serum Albumin Redox State Is Associated with Infant Birth Weight in Japanese Pregnant Women

**DOI:** 10.3390/nu13061764

**Published:** 2021-05-22

**Authors:** Yasuaki Wada, Tatsuya Ehara, Fuka Tabata, Yosuke Komatsu, Hirohisa Izumi, Satomi Kawakami, Kiwamu Noshiro, Takeshi Umazume, Yasuhiro Takeda

**Affiliations:** 1 Wellness & Nutrition Science Institute, Morinaga Milk Industry Co., Ltd., 5-1-83 Higashihara, Zama, Kanagawa 252-8583, Japan; t-ehara@morinagamilk.co.jp (T.E.); fuuka-tabata131@morinagamilk.co.jp (F.T.); yo-komatsu@morinagamilk.co.jp (Y.K.); h-izumi@morinagamilk.co.jp (H.I.); s_kawakm@morinagamilk.co.jp (S.K.); ya_taked@morinagamilk.co.jp (Y.T.); 2 Center for Food and Medical Innovation Promotion, Institute for the Promotion of Business-Regional Collaboration of Hokkaido University, Kita-21, Nishi-11, Kita-ku, Sapporo, Hokkaido 001-0021, Japan; 3 Department of Obstetrics and Gynecology, Hokkaido University Graduate School of Medicine, Kita-15, Nishi-7, Kita-ku, Sapporo, Hokkaido 060-8648, Japan; noshiro.kiwamu@pop.med.hokudai.ac.jp (K.N.); takeuma@med.hokudai.ac.jp (T.U.)

**Keywords:** albumin, protein-energy restriction, fetal growth restriction, low birth weight delivery, serum albumin redox state

## Abstract

Background: Plasma albumin (ALB) reflects protein nutritional status in rats, but it is not clear whether it is associated with dietary protein insufficiency in pregnant women and/or their risk of low birth weight delivery. This study aimed to investigate whether maternal serum ALB redox state reflects maternal protein nutritional status and/or is associated with infant birth weights. Methods: The relationship between the serum reduced ALB ratio and infant birth weight was examined in an observational study of 229 Japanese pregnant women. A rat model simulating fetal growth restriction, induced by protein-energy restriction, was used to elucidate the relationship between maternal nutritional status, maternal serum ALB redox state, and birth weight of the offspring. Results: In the human study, serum reduced ALB ratio in the third trimester was significantly and positively correlated with infant birth weight. In the rat study, serum reduced ALB ratio and birth weight in the litter decreased as the degree of protein-energy restriction intensified, and a significant and positive correlation was observed between them in late pregnancy. Conclusions: Maternal serum reduced ALB ratio in the third trimester is positively associated with infant birth weight in Japanese pregnant women, which would be mediated by maternal protein nutritional status.

## 1. Introduction

Low birth weight (LBW) delivery is defined as a delivery in which infant birth weight is <2500 g regardless of gestational age [1]. LBW delivery is largely attributed to premature birth and/or fetal growth restriction (FGR) in utero. Pre-pregnant women who are underweight (body mass index (BMI) <18.5 kg/m^2^) and/or those who have insufficient gestational weight gain are at high risk of LBW delivery resulting from fetal undernutrition [2,3,4,5,6,7], and maternal dietary pattern and nutritional status are the pivotal factors specifying the fate of delivery [3,8]. LBW delivery is highly related to neonatal mortality and morbidity [1], and fetal undernutrition can even inflict epigenetically negative impacts on the fetus, thereby increasing the risk of non-communicative diseases later in life [9]. This latter notion is known as the “developmental origins of health and disease (DOHad)” hypothesis [10], which has been robustly demonstrated by animal studies where dams were given low-protein diets [11,12,13], or maintained under energy-restricted conditions [14,15,16,17]. Notably, the incidence of LBW infants is overtly higher in Japan (9.4%) compared with the average of Organization for Economic Cooperation and Development (OECD) countries (6.5%) [6]. The incidence of LBW infants in Japan has increased during the last three decades [18], which is presumably associated with the “desire for thinness” rooted in Japanese women at a reproductive age [19]. Specifically, the mean energy intakes of Japanese pregnant women were below the estimated average requirement (EAR) of the Japanese dietary reference intakes (DRIs) in all of the three trimesters, and their mean protein intake was also lower than the recommended daily allowance (RDA) in the third trimester [3,20].

Albumin (ALB) is the most abundant circulating protein [21]. ALB can be categorized into three isoforms according to the redox states of the cysteine residue at position 34 (Cys34), i.e., reduced ALB, oxidized ALB-1, and oxidized ALB-2 [22]. Oxidized shifts of serum ALB redox state have been confirmed in oxidative stress-related diseases such as liver diseases and renal failure, and ALB redox state has, therefore, been viewed as an oxidative stress marker [22]. However, recent animal studies have shown that the ALB redox state was shifted to a more oxidized state in rats fed low-protein diets [23,24,25]. This shift was mediated by decreased ALB turnover, and was independent of oxidative stress in these low-protein diet models [23,25]. Moreover, ALB redox state responded to the low-protein diet ingestion more sensitively than conventional protein nutrition biomarkers such as the concentrations of ALB and transthyretin [24,25]. Thus, the ALB redox state could help identify pregnant women that are potentially undernourished and/or are at higher risk of LBW delivery. There has been one report in which reduced ALB was decreased in pregnant women diagnosed with FGR during pregnancy compared with healthy subjects [26]. Still, the associations between ALB redox state and birth outcome per se, i.e., infant birth weight, have not yet been confirmed yet.

We hypothesized serum maternal ALB redox state during pregnancy would be correlated with infant birth weight, which could be mediated by maternal nutritional status. The aim of this study was to investigate whether maternal serum ALB redox state is associated with maternal nutritional status and/or infant birth weights. We conducted an observational study on Japanese pregnant women to examine the association between serum ALB redox state and infant birth weight. We also executed an animal experiment that simulated FGR, by maintaining pregnant rats on an AIN-93G diet [27] ad libitum or on the same diet under energy-restricted conditions (i.e., protein-energy-restricted condition), and the relationship between serum ALB redox state of the dams during pregnancy and birth weight of the pups were examined.

## 2. Materials and Methods

### 2.1. Human Observational Study

This study was conducted in accordance with the principles of the Declaration of Helsinki and with the approval of the Institutional Review Board of Hokkaido University Hospital (Protocol No. 017-0281). Pregnant women that were scheduled to give birth between October 2018 and April 2019 were recruited at Obihiro Kosei General Hospital, Hakodate Central General Hospital, and JCHO Hokkaido Hospital, all of which are located in Hokkaido prefecture, Japan. Women were eligible if they (1) were aged 18 or older at recruitment, (2) intended to receive medical examinations during pregnancy and to give birth at any of the above three hospitals, and (3) were well informed of, in full understanding of, and in agreement with their participation in the study. Exclusion criteria were the women (1) had their first medical examination at 14 weeks of gestation or later and (2) were judged as inappropriate to participate in this study by the physicians. A total of 379 women participated in this study, and written informed consent was obtained from all of them. The participants visited the hospitals for medical examinations during the first (<14 weeks of gestation), the second (between 24 weeks and 27 weeks + 6 days of gestation), and the third trimester (around 36 weeks of gestation) of the gestational period. During the visits, demographic information was collected; blood was drawn from the antecubital vein and the serum fraction was obtained after centrifugation and stored at −80 °C. Demographic information was also collected at delivery.

**Figure 1:**
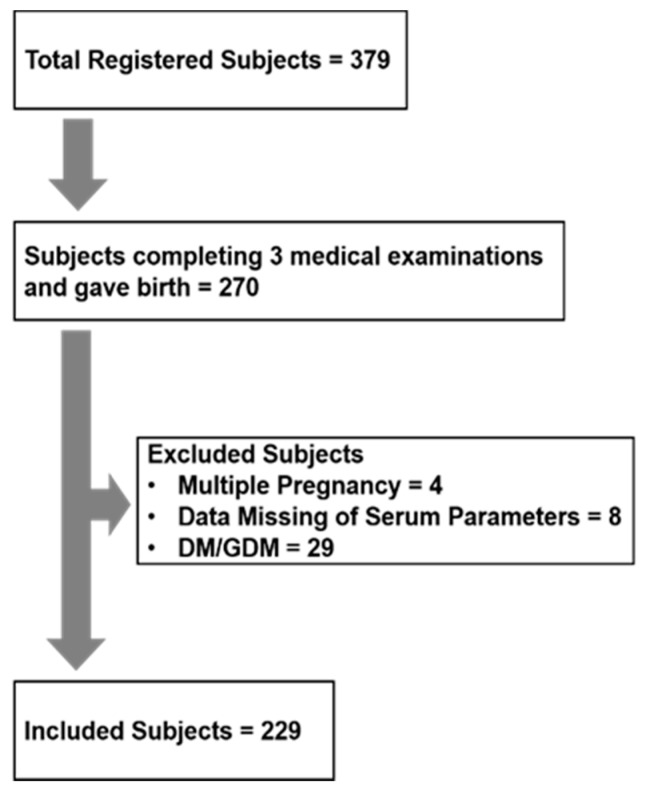
Flow chart for selecting subjects. A total of 379 subjects participated in this study, and 270 completed all three medical examinations during pregnancy, and gave birth. Four subjects were excluded as they had multiple pregnancies, 8 subjects were excluded as they had missing data on blood biochemistry, and 29 subjects were excluded as they were diagnosed with diabetes mellitus or gestational diabetes mellitus (DM/GDM). The remaining 229 participants were included in the analyses, and none of these participants were diagnosed with oxidative stress-related diseases such as liver diseases and renal failure.

This study was a part of a larger study; it was conceived and the approval was obtained again after the remaining part was completed. All the analyses were thus conducted retrospectively. Among the 379 participants, 270 completed all three medical examinations during pregnancy, and gave birth. For the analyses of this study, 4 subjects were excluded as they had multiple pregnancies, 8 subjects were excluded as they had missing data on blood biochemistry, and 29 subjects were excluded as they were diagnosed with diabetes mellitus or gestational diabetes mellitus (DM/GDM); DM is a risk factor for large for gestational age rather than premature delivery [28], and DM/GDM per se affects serum ALB redox state [22]. The remaining 229 participants were included in the analyses (Figure 1). None of these participants were diagnosed with oxidative stress-related diseases such as liver diseases and renal failure.

### 2.2. Animal Experiment Study

The design of this animal experiment was proposed by the authors, and then approved by the Animal Research Committee of Morinaga Milk Industry Co., Ltd. (Protocol No. 18-026). The experiment was performed between September 13th and October 4th, 2018, in accordance with the committee’s guidelines. Eighteen pregnant Wistar rats (dams) on pregnancy day1 (PD1) were commercially obtained (Japan SLC, Hamamatsu, Japan). The dams were acclimated to the facility condition for 8 days [23,24,25], during which they were allowed ad libitum access to water and the AIN-93G diet (Oriental Yeast, Tokyo, Japan [27]). On PD10, the dams were assigned to 3 dietary groups (*n* = 6), being fed the AIN-93G diet ad libitum, or given the same diet reduced by 20%, or 40% of the average amount of ad libitum feeding. These groups were designated as the control (CTRL), 20% energy restriction (20% ER), and 40% energy restriction (40% ER) groups, respectively, and were maintained on this dietary regimen from PD10 until delivery (PD22). On PD9, PD16, and PD19, body weights of the dams were measured, and blood was collected from the lateral tail vein on the same days. The blood samples were then centrifuged, and the serum fraction was obtained after centrifugation and stored at −80 °C. On the delivery day (PD22), body weights of all of the pups were measured. The sum of birth weights in each dam was designated as “litter weight”, and the litter weight was then divided by the litter size (number of pups) to obtain the “birth weight in the litter”. All of the dams and pups were finally euthanized by deep anesthesia with sevoflurane (Mylan, Pittsburgh, PA, USA).

### 2.3. Blood Biochemistry

Serum ALB redox state was expressed by the ratio of reduced to total ALB, designated as serum reduced ALB ratio; it was determined by applying serum samples to HPLC as described previously [25,29]. As serum ALB oxidation could potentially proceed further in clinical sites due to its high susceptibility to ambient temperature > –80 °C [30], the serum reduced ALB ratio was also determined for the rat samples obtained on PD19 after the oxidation was intentionally facilitated, namely, by incubating aliquots of rat serum samples on PD19 at 4 °C for 5 weeks. Serum ALB concentration was determined using a bromocresol green method (an A/G B test kit, Wako Pure Chemical, Osaka, Japan). Serum BUN concentration was determined by using a BUN colorimetric detection kit (Arbor Assays, Ann Arbor, MI, USA).

### 2.4. Statistical Analyses

Data were analyzed by one-way ANOVA followed by a Tukey–Kramer HSD test, simple linear regression analyses, and multiple linear regression analyses using JMP software (version 13.2.1; SAS Institute, Cary, NC, USA). Significance was demonstrated at *p* < 0.05. As for the animal experiment, a wild type animal model was selected in this experiment and statistical analyses were conducted based on the assumption that the dietary groups had normal distributions and homogeneity of variance. As for multiple linear regression analyses vs. infant birth weight, the serum reduced ALB ratio in the third trimester, pre-pregnancy body weight, pre-pregnancy BMI, gestation period, and body weight at delivery, as reported in the Results section, were selected as independent variables as they were found to be significantly correlated with infant birth weight in single linear regression analyses (*p* < 0.05).

## 3. Results

### 3.1. Human Observational Study

#### 3.1.1. Characteristics of participants

Characteristics of the participants are summarized in Table 1. Among the 229 participants analyzed in this study, 40 participants had pre-pregnancy BMI < 18.5 (17.5%). The mean ± SD of infant birth weight was 3107 ± 388 g, and 9 and 10 of 229 infants had LBW delivery (3.9%) and preterm delivery (4.4%), respectively.

#### 3.1.2. Blood Biochemistry in Pregnant Women in the First, Second, and Third Trimesters

**Figure 2:**
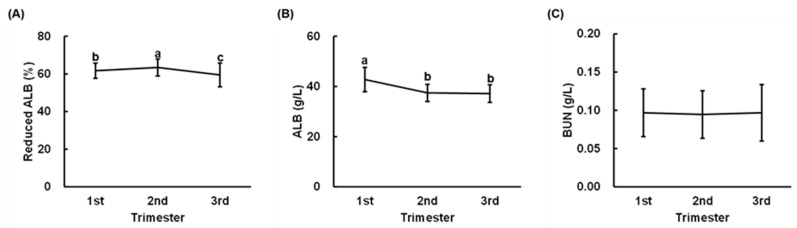
Serum reduced albumin (ALB) ratio, ALB concentration, and blood urea nitrogen (BUN) concentration in pregnant women. Serum samples of pregnant women, obtained in the first-third trimester, were applied to the analyses of reduced ALB ratio (**A**), ALB concentration (**B**), and BUN concentration (**C**). Data are expressed as means ± SDs (*n* = 229), which were analyzed by one-way ANOVA followed by a Tukey–Kramer HSD test. Values with different letters are significantly different (*p* < 0.05).

Serum reduced ALB ratios differed significantly between the trimesters; the ratio in the second trimester was the highest and that of the third trimester was the lowest (Figure 2A). Serum ALB concentrations in the second and third trimesters were significantly lower than that of the first trimester, but the concentrations of the second and third trimesters did not differ significantly (Figure 2B). No significant difference in the serum BUN concentration was seen between the trimesters (Figure 2C).

#### 3.1.3. Relationship between Maternal Parameters and Infant Birth Weight 

Simple linear regression analyses were conducted to examine the relationships of maternal serum parameters vs. infant birth weight (Table 2). Serum reduced ALB ratio in the third trimester was the only serum parameter that was significantly correlated with infant birth weight. Simple linear regression analyses were also conducted to examine the relationships of maternal background/gestational outcome vs. infant birth weight (Table S1). Parameters that correlated significantly with infant birth weight were pre-pregnancy body weight, pre-pregnancy BMI, gestation period, and body weight at delivery. Simple linear regression analyses were further investigated for any pairs of the above five parameters (Table S2). Serum reduced ALB ratio in the third trimester was significantly correlated with gestation period only, and the correlation was positive. Significant correlations were also observed in (1) pre-pregnancy body weight vs. pre-pregnancy BMI, (2) pre-pregnancy body weight vs. body weight at delivery, and (3) pre-pregnancy BMI vs. body weight at delivery. Single linear regression analyses of maternal serum parameters vs. infant birth weight were also conducted in mother/infant dyads in the first quartile of infant birth weight (n = 58), where LBW delivery comprised 15.5% (9/58). It was found, however, that the relationship between maternal serum reduced ALB ratio in the third trimester and infant birth weight did not reach statistical significance despite a similar coefficient R observed (R = 0.198 and *p* = 0.138), presumably because of the small sample size.

Multiple linear regression analyses were then conducted for combinations of the above five parameters vs. infant birth weight (Table 3). Serum reduced ALB ratio in the third trimester was significantly and positively correlated with infant birth weight when one of the three parameters, pre-pregnancy body weight, pre-pregnancy BMI, and body weight at delivery was selected as another independent variable (model 1–3), while no significant correlation was seen between the serum reduced ALB ratio and infant birth weight when gestation period was selected as another independent variable (model 4). Similarly, the correlations were not significant between the serum reduced ALB ratio and infant birth weight when gestation period was added to the model 1-3 as an independent variable (model 5–7).

### 3.2. Animal Experiment

#### 3.2.1. Animal Characteristics

The average energy and protein intakes of the dams were 196.2 kJ/d and 2.2 g/d in the CTRL group, 143.1 kJ/d and 1.6 g/d in the 20% ER group, and 107.7 kJ/d and 1.2 g/d in the 40% ER group, respectively. Body weights of the 40% ER group were significantly lower compared with the CTRL group on PD16 and PD19, whereas weight gains were different between all the three groups on PD16 and PD19 in the same directionality as the energy/protein intakes (Figure S1). When the dams delivered, litter sizes and weights did not differ significantly between the groups, while birth weight in the litter was the lowest in the 40% ER group and significantly lower than that of the CTRL group (Table S3).

#### 3.2.2. Blood Biochemistry in Pregnant rats on PD9, PD16, and PD19

**Figure 3:**
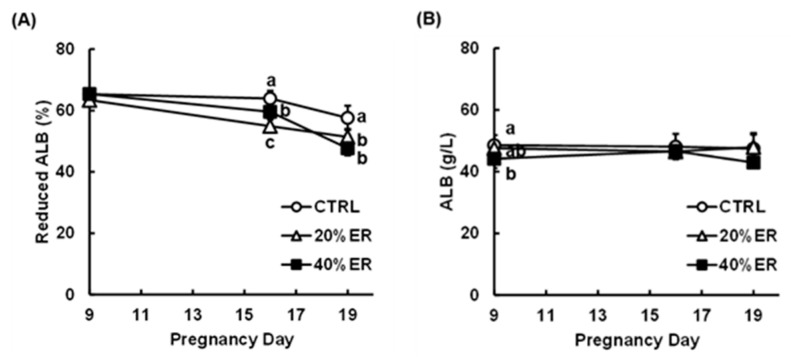
Serum reduced albumin (ALB) ratio and serum ALB concentration in pregnant rats. Serum samples of pregnant rats, obtained on pregnancy day 9 (PD9), 16, and 19, were applied to the analyses of reduced ALB ratio (**A**) and ALB concentration (**B**). Data are expressed as means ± SDs (*n* = 6), which were analyzed by one-way ANOVA followed by a Tukey–Kramer HSD test; at each time point, values with different letters are significantly different (*p* < 0.05). CTRL, control; 20% ER, 20% energy restriction; 40% ER, 40% energy restriction.

Serum reduced ALB ratios of the 20% ER and 40% ER groups were significantly lower compared with the CTRL group on both PD16 and 19 (Figure 3A). In contrast, no significant difference in the serum ALB concentration was seen between the groups on PD16 and PD19 (Figure 3B), although serum ALB concentrations differed significantly between the CTRL and the 40% ER group on PD9 (i.e., before starting the dietary regimen).

#### 3.2.3. Relationship between Maternal Serum Parameters and Birth Weight in the Litter.

Simple linear regression analyses were conducted to examine the relationship of the serum reduced ALB ratio or ALB concentration vs. birth weight in the litter; the analyses were conducted for all the animals in the three dietary groups together (Table 4). Serum reduced ALB ratio on PD19 was significantly and positively correlated with birth weight in the litter. Although a significant correlation was seen between serum ALB concentration on PD9 (i.e., before starting the dietary regimen) and birth weight in the litter, no significant correlation was seen between serum ALB concentration on PD16 or 19 and birth weight in the litter.

When serum samples on PD19 were incubated at 4 °C for 5 weeks, the means ± SDs of reduced ALB ratios were down to 36.3 ± 6.7% in the CTRL group, 33.1 ± 5.4% in the 20% ER group, and 28.5 ± 3.9% in the 40% ER group, respectively, and significant differences in the serum reduced ALB ratio seen between the groups before the incubation was dissolved after the incubation. Still, the significant and positive correlation between the serum reduced ALB ratio and birth weight in the litter was persistent after the incubation (Table 4).

## 4. Discussion

Protein-energy undernutrition is considered potentially prevalent in Japanese pregnant women [3,20], and they are therefore at a higher risk of LBW delivery compared with those in other developed countries [6]. With an emphasis on the potential usefulness of serum ALB redox state as a nutrition biomarker [23,24,25], the present study investigated whether serum ALB redox state during pregnancy is associated with infant birth weight in Japanese pregnant women as well as in a rat model simulating FGR by protein-energy restriction. 

In the human observational study, LBW delivery comprised 3.9% of all the participants, which is lower than previously reported for Japanese pregnant women (9.4%) [6]. Among the maternal serum parameters of protein nutritional status examined in this study, simple linear regression analyses showed that serum reduced ALB ratio in the third trimester was the only serum parameter that was significantly correlated with infant birth weight, and the correlation was positive. Four of the maternal background/gestational outcome parameters, pre-pregnancy body weight, pre-pregnancy BMI, body weight at delivery, and gestation period, were also correlated significantly with infant birth weight in the simple linear regression analyses. Multiple linear regression analyses were then conducted for combinations of the above five parameters vs. infant birth weight. Serum reduced ALB ratio was significantly and positively correlated with infant birth weight when pre-pregnancy body weight, pre-pregnancy BMI, or body weight at delivery, was included as another independent variable. In contrast, no significant correlation was seen between serum reduced ALB ratio and infant birth weight when gestation period was selected as another independent variable, which was attributed to the observation that serum reduced ALB ratio in the third trimester was correlated significantly and positively with gestation period. Thus, serum reduced ALB ratio in the third trimester was significantly and positively associated with infant birth weight; the association was independent of maternal anthropometry (pre-pregnancy body weight/BMI and body weight at delivery), but the involvement of gestation period was implicated. While it is reasonable that a shorter gestation period would be accompanied by a lower birth weight delivery [1], the mechanisms that associate serum reduced ALB ratio with gestation period remain to be clarified. A recent systematic review reported a beneficial effect of high protein intake on a reduction in the risk of preterm birth [8], and recent studies in rats showed that plasma reduced ALB ratio decreased sensitively in response to dietary protein insufficiency [23,24,25]. Although the dietary assessment was not conducted, the association between serum reduced ALB ratio in the third trimester and infant birth weight/gestation period observed in this study might reflect the considerable prevalence of maternal dietary protein insufficiency in the third trimester, as observed previously in the Japanese population [3,20]. However, the effects of protein intake during pregnancy on FGR and LBW delivery have not been fully elucidated. Namely, meeting the minimum protein requirement may be helpful to prevent the risk of FGR [31,32,33,34,35], while a higher protein intake may do more harm than good [32,35,36]. It has been proposed that there is a quadratic (inverse U-curve) relationship between maternal protein intake and fetal growth [35,37], and moderate protein intake with an appropriate energy/protein balance is required to optimize fetal growth [35,38]. Thus, as well as protein intake, the quantity of dietary carbohydrate and fat needs to be considered for better gestational outcomes. Notably, higher intake of n-3 polyunsaturated fatty acids or lower intake of added sugars was positively associated with gestational period and/or infant birth weight [8], indicating that the “quality” of macronutrients is influential for gestational outcomes. Lastly, a recent study on healthy pregnant women in Canada reported that the protein requirement could be higher than the current DRI recommendation [39]. The same research group subsequently showed increased requirements of specific indispensable amino acids, such as lysine and phenylalanine, during pregnancy [40,41]. Thus, caution may be necessary to view the risk of FGR and LBW in the context of maternal protein intake.

The relationship between serum ALB redox state and birth weight of the offspring was also examined in an FGR model in rats. In order to simulate FGR, pregnant animals are normally placed on low-protein diets [11,12,13], or maintained under protein-energy restriction [14,15,16,17]. Protein-energy restriction was selected here in light of the above-mentioned observations that Japanese pregnant women would have insufficient energy intake as well as protein intake [3,20]. Weight gains of the dams on both PD16 and 19 differed significantly between the groups, which demonstrated the same directionality to their energy intakes. Similarly, birth weight in the litter was the lowest in the 40% ER group and significantly lower compared with the CTRL group, and the 20% ER group had the intermediate mean birth weight in the litter between the CTRL and 40% ER groups. Thus, the protein-energy restriction of this study successfully brought about FGR and the resulting lower birth weight delivery in a protein-energy-dose-dependent manner. Serum ALB concentrations did not differ significantly between the groups on either PD16 or 19, while serum reduced ALB ratios of the two energy-restricted groups were significantly lower compared to the CTRL group on PD16 and 19. This observation was in agreement with previous reports that plasma ALB redox state was more responsive to lower dietary protein intake compared with ALB concentrations in young and adult rats [23,24,25]. Furthermore, serum reduced ALB ratio in PD19 was the only serum parameter examined in the study that was significantly correlated with birth weight in the litter. Taken together, serum reduced ALB ratio decreased in response to maternal protein insufficiency as early as mid-pregnancy (on PD16), and the decreased ratio in late-pregnancy (on PD19) was eventually correlated significantly with the resulting lower birth weight. Thus, maternal serum ALB redox state would be associated with the birth weight of the offspring mediated by maternal protein nutritional status, as we hypothesized.

The largest limitation of this study was that, when considering the fact that serum reduced ALB ratio is normally >70% [22], albumin oxidation was apparently facilitated in all of the human serum samples. As mentioned in the Materials and Methods section, this human observational study is part of a larger study (unpublished), and it took several months from serum sample collection until the analysis of reduced ALB ratio, during which serum samples were thawed and re-frozen several times for the other parts of the entire study. Due to the high susceptibility of serum ALB to further oxidation at ambient temperature >−80 °C [30], ALB oxidation would thus be facilitated in the handling. In consideration of this, ALB oxidation was intentionally facilitated for rat serums, and it was observed that the significant and positive correlation between the serum reduced ALB ratio and birth weight in the litter was persistent even after ALB oxidation was facilitated, although the coefficient correlation R was decreased by the oxidation. When extrapolating this observation to the human observational study, it can be interpreted that the associations between the serum reduced ALB ratio and infant birth weight would be attenuated by increased ALB oxidation in the handling, which may obscure the true potential of serum reduced ALB ratio as an indicator for the risk of LBW delivery. Another limitation of this study may be that one hospital among the three adopted only fasting serum glucose concentration for the diagnosis of GDM, instead of using an oral glucose tolerance test, while the other two hospital adopted oral glucose tolerance tests. Exclusion of subjects with GDM might have been insufficient in the present study, which could also have attenuated the association between serum reduced ALB ratio and infant birth weight [22,28]. Regarding the limitation of the animal study, while the involvement of gestation period was implicated for the correlation between serum reduced ALB ratio and infant birth weight in the human observational study, it was not able to be evaluated in this animal study because of their very short gestational period (all the dams delivered on PD22). Gestational periods of rats are, thus, quite short and it was quite difficult to discern the difference in the length between animals. Further clinical studies are required to elucidate the potential association between the serum ALB redox state and infant birth weight, mediated by gestational period.

In conclusion, maternal serum reduced ALB ratio in the third trimester is associated with infant birth weight in Japanese pregnant women, which is also substantiated by a rat model simulating FGR by protein-energy restriction, as we hypothesized. According to the rat model and with reference to previous literature [23,24,25], protein nutritional status is likely involved in the association between maternal serum ALB redox state and birth weight of the offspring. Clinical studies that involve dietary assessment and/or dietary intervention are required to substantiate the association between maternal nutritional status, serum ALB redox state, and birth weight of the offspring.

## Figures and Tables

**Table 1:** Characteristics of participants.

Characteristics	
Maternal background	
Participants (n)	229
Primipara (n)	83 (36.2%)
Age (years)^*1^	31.6 ± 5.0
Height (cm)^*1^	158.1 ± 5.4
Pre-pregnancy body weight (kg)^*1 & 2^	51.7 ± 9.7
Pre-pregnancy BMI^*2^	21.1 ± 3.4
Pre-pregnancy BMI < 18.5 (n) ^*2^	40 (17.5%)
Gestational outcome	
Gestation period (weeks)^*1^	39.2 ± 1.2
Body weight at delivery (kg)^*1^	63.9 ± 9.9
Weight gain (kg)^*1^	11.0 ± 4.0
Vaginal delivery, Caesarean section (n, n)	182, 47
Male, Female (n, n)	116, 113
Birth weight (g)^*1^	3107 ± 388
Low birth weight delivery (<2500 g; n)	9 (3.9%)
Preterm delivery (<37 weeks; n)	10 (4.4%)

^*1^ Means ± SDs. ^*2^ Obtained by a questionnaire at the first clinical visit. BMI, body mass index.

**Table 2:** Simple linear regression analyses of serum biochemistry vs. infant birth weight in pregnant women.

Independent Variable	R	*p*
Serum reduced ALB ratio		
1st trimester	0.066	0.322
2nd trimester	0.108	0.105
3rd trimester	0.177	<0.01
Serum ALB concentration		
1st trimester	0.095	0.154
2nd trimester	0.009	0.888
3rd trimester	−0.066	0.325
Serum BUN concentration		
1st trimester	0.069	0.296
2nd trimester	0.024	0.718
3rd trimester	−0.011	0.864

ALB, albumin; BUN, blood urea nitrogen.

**Table 3:** Multiple linear regression analyses of maternal parameters vs. infant birth weight in pregnant women^*1^.

	Independent Variable	Coefficient	SE	β	VIF	*p*
Model 1	(Intercept)	1930.68	281.31	−		<0.0001
R^2^ = 0.079 *p* < 0.0001	Serum reduced ALB^*2^	12.02	3.94	0.196	1.01	<0.01
	Pre-pregnancy body weight	8.72	2.57	0.218	1.01	<0.001
Model 2	(Intercept)	1978.40	288.32	−		<0.0001
R^2^ = 0.06 *p* <0.001	Serum reduced ALB^*2^	11.33	3.95	0.184	1.00	<0.01
	Pre-pregnancy BMI	21.54	7.42	0.187	1.00	<0.01
Model 3	(Intercept)	1794.11	285.69	−		<0.0001
R^2^ = 0.095 *p* < 0.0001	Serum reduced ALB^*2^	11.39	3.89	0.185	1.00	<0.01
	Body weight at delivery	9.94	2.49	0.253	1.00	<0.0001
Model 4	(Intercept)	−2911.12	766.32	−		<0.001
R^2^ = 0.216 *p* < 0.0001	Serum reduced ALB^*2^	5.60	3.69	0.091	1.04	0.131
	Gestation period	145.16	19.87	0.439	1.04	<0.0001
Model 5	(Intercept)	−3186.05	755.99	-		<0.0001
R^2^ = 0.251 *p* < 0.0001	Serum reduced ALB^*2^	6.73	3.63	0.110	1.05	0.065
	Pre-pregnancy body weight	7.45	2.33	0.186	1.01	<0.01
	Gestation period	140.40	19.54	0.424	1.05	<0.0001
Model 6	(Intercept)	−3186.05	755.99	−		<0.0001
R^2^ = 0.240 *p* < 0.001	Serum reduced ALB^*2^	6.10	3.64	0.099	1.04	0.096
	Pre-pregnancy BMI	17.92	6.73	0.155	1.01	<0.01
	Gestation period	141.25	19.67	0.427	1.05	<0.0001
Model 7	(Intercept)	−3180.65	750.21	−		<0.0001
R^2^ = 0.260 *P-p* < 0.0001	Serum reduced ALB^*2^	6.28	3.60	0.102	1.04	0.082
	Body weight at delivery	8.22	2.27	0.209	1.01	<0.001
	Gestation period	137.59	19.47	0.416	1.05	<0.0001

^*1^ Dependent variable: infant birth weight (g); independent variables: serum reduced ALB ratio (%), pre-pregnancy body weight (kg), pre-pregnancy BMI, body weight at delivery (kg), and/or gestation period (week). ^*2^ Analyzed in the third trimester. ALB, albumin; BMI, body mass index; VIF, variance inflation factor.

**Table 4:** Simple linear regression analyses of serum reduced albumin (ALB) ratio and serum ALB concentration vs. birth weight in the litter in pregnant rats^*1^.

Independent Variable	R	*p*
Reduced ALB		
PD9	−0.102	0.687
PD16	0.198	0.432
PD19	0.697	<0.01
PD19inc	0.614	<0.01
ALB		
PD9	0.514	<0.05
PD16	0.381	0.119
PD19	0.294	0.237

^*1^ Analyses were conducted for all the animals in the three dietary groups together (18 animals). ALB, albumin; PD, pregnancy day; inc, incubation (4 °C × 5 weeks).

## Supplementary Materials

The following are available online at https://www.mdpi.com/article/10.3390/nu13061764/s1, Figure S1: Body weights and weight gain of pregnant rats, Table S1: Simple linear regression analyses of maternal background/gestational outcome vs. infant birth weight in pregnant women, Table S2: Simple linear regression analyses between serum ALB redox state in the third trimester, pre-pregnancy body weight, pre-pregnancy BMI, gestation period, and body weight and delivery, and Table S3: Birth outcomes of pregnant rats.
