# Supplementary material for: Maternal Serum Albumin Redox State Is Associated with Infant Birth Weight in Japanese Pregnant Women
Source: Nutrients. 2021 May 22;13(6):1764. doi: 10.3390/nu13061764 (PMC8224550; doi:10.3390/nu13061764)
Supplement: Supplementary file 1 [file nutrients-13-01764-s001.zip › nutrients-1201156-supplementary.pdf]

Figure S1 Body weights and weight gain of pregnant rats.

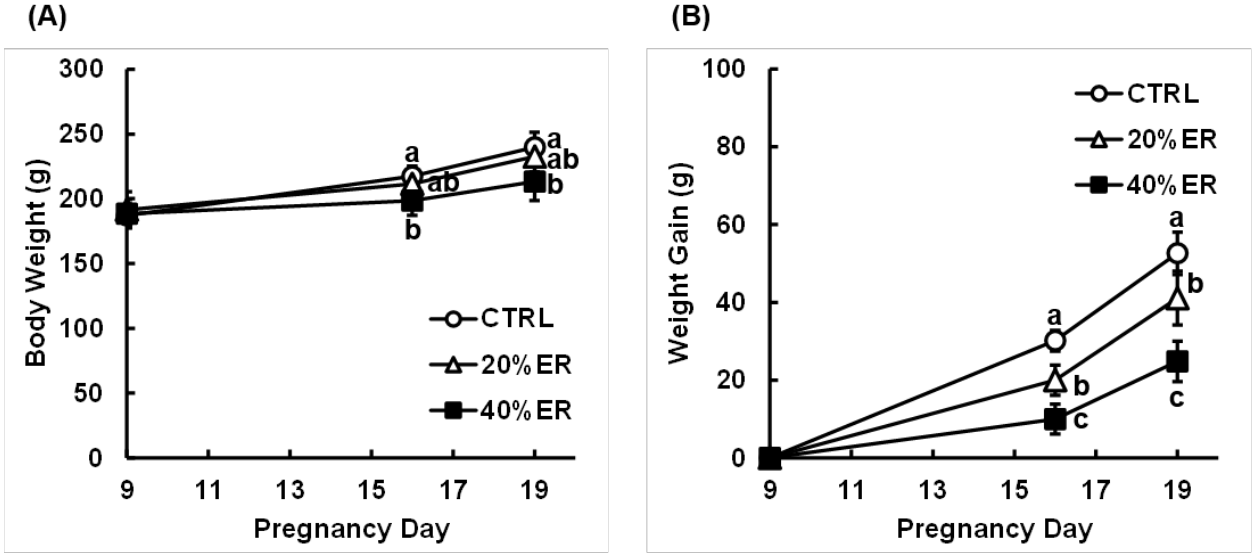

Body weights (a) and weight gain (b) of pregnant rats were measured on the pregnancy day 9 (PD9), 16, and 19. Weight gains during the experimental period were also calculated. Data are expressed as means ± SDs (n = 6), which were analyzed by one-way ANOVA followed by a Tukey-Kramer HSD test; at each time point, values with different letters are significantly different ($p < 0.05$). CTRL, control; 20%ER, 20% energy restriction; 40%ER, 40% energy restriction.

Table S1 Simple linear regression analyses of maternal background/gestational outcome vs infant birth weight in pregnant women.

Independent Variable	R	*p*
Maternal background		
Age	−0.028	0.677
Height	0.119	<0.073
Pre-pregnancy body weight	0.201	<0.01
Pre-pregnancy BMI	0.180	<0.01
Gestational Outcome		
Gestation Period	0.457	<0.0001
Body weight at delivery	0.247	<0.001
Weight gain	0.121	0.068

BMI, body mass index.

Table S2 Simple linear regression analyses between serum ALB redox state in the third trimester, pre-pregnancy body weight, pre-pregnancy BMI, gestation period, and body weight and delivery in pregnant women.

	Serum ALB redox state[*1]	Pre-pregnancy body weight	Pre-pregnancy BMI	Gestation Period	Body weight at delivery
Serum ALB redox state[*1]	-	R = −0.084<br>$p = 0.207$	R = −0.037<br>$p = 0.573$	R = 0.197<br>$p < 0.01$	R = −0.032<br>$p = 0.634$
Pre-pregnancy body weight	R = −0.084<br>$p = 0.207$	-	R = 0.929<br>$p < 0.0001$	R = 0.058<br>$p = 0.384$	R = 0.915<br>$p < 0.0001$
Pre-pregnancy BMI	R = −0.037<br>$p = 0.573$	R = 0.929<br>$p < 0.0001$	-	R = 0.066<br>$p = 0.321$	R = 0.827<br>$p < 0.0001$
Gestation Period	R = 0.197<br>$p < 0.01$	R = 0.058<br>$p = 0.384$	R = 0.066<br>$p = 0.321$	-	R = 0.099<br>$p = 0.136$
Body weight at delivery	R = −0.032<br>$p = 0.634$	R = 0.915<br>$p < 0.0001$	R = 0.827<br>$p < 0.0001$	R = 0.099<br>$p = 0.136$	-

[*1] Analyzed in the third trimester.

ALB, albumin; BMI, body mass index.

Table S3 Birth outcomes of pregnant rats.

Outcome	CTRL	20% ER	40% ER
Litter weight (g)	40.2 ± 15.6	45.3 ± 8.6	40.5 ± 7.7
Litter size	8.3 ± 3.3	10.0 ± 2.2	9.7 ± 2.3
Birth weight in the litter	4.9 ± 0.2[a]	4.6 ± 0.2 [ab]	4.2 ± 0.3 [b]

Data are expressed as means ± SD (n = 6), which were analyzed by one-way ANOVA followed by a Tukey-Kramer HSD test; values with different letters are significantly different ($p < 0.05$). CTRL, control; 20%ER, 20% energy restriction; 40%ER, 40% energy restriction.
